# Estimating the annual burden of tick-borne encephalitis to inform vaccination policy, Slovenia, 2009 to 2013

**DOI:** 10.2807/1560-7917.ES.2017.22.16.30509

**Published:** 2017-04-20

**Authors:** Mario Fafangel, Alessandro Cassini, Edoardo Colzani, Irena Klavs, Marta Grgič Vitek, Veronika Učakar, Marion Muehlen, Marko Vudrag, Alenka Kraigher

**Affiliations:** 1 National Institute of Public Health (NIJZ), Ljubljana, Slovenia; 2 European Programme for Intervention Epidemiology Training (EPIET), European Centre for Disease Prevention and Control (ECDC), Stockholm, Sweden; 3 European Centre for Disease Prevention and Control (ECDC), Stockholm, Sweden; 4 Julius Centre for Health Sciences and Primary Care, University Medical Centre Utrecht, Utrecht, The Netherlands; 5 Department of Health Science, University of Milano-Bicocca, Monza, Italy

**Keywords:** burden of illness, tick-borne encephalitis - TBE, viral meningitis, viral encephalitis, vaccination

## Abstract

With an annual incidence between 8 and 15 per 100,000 population in the period from 2009 to 2013, Slovenia has one of the highest notified incidences of tick-borne encephalitis (TBE) in Europe. TBE vaccination coverage remains at about 7.3%. To inform vaccination policy, we used surveillance data from 2009 to 2013 to calculate the overall and age- and sex-specific mean annual TBE incidence. We estimated disability-adjusted life years (DALYs) with 95% uncertainty intervals (UI), using the Burden of Communicable Diseases in Europe approach from the European Centre for Disease Prevention and Control. The mean annual incidence was 11.6 per 100,000 population, peaking in older age groups (50–74 years: 18.5/100,000) while relatively lower among children (5–14 years: 10.2/100,000). We estimated an overall 10.95 DALYs per 100,000 population per year (95% UI: 10.25-11.65). In contrast to the TBE incidence, the disease burden in children aged 5–14 years was higher than in adults aged 50–74 years: 17.31 (95% UI: 14.58–20.08) and 11.58 (95% UI: 10.25–12.91) DALYs per 100,000 stratum-specific population, respectively. In a limited resource setting where prioritisation of TBE vaccination strategies is required, vaccination programmes targeting children may have a higher impact on disease burden.

## Introduction

Tick-borne encephalitis (TBE) is a vector-borne disease caused by the TBE virus [1]. It typically presents as a two-phased illness [2-4]. The first phase is associated with symptoms such as fever, fatigue, headache, myalgia and nausea. The second phase involves the nervous system with symptoms related to meningitis and/or encephalitis. Life-long sequelae can have an important impact on the quality of life of those affected [5]. TBE cases notified in Europe have surged in the last three decades with an estimated increase of 193% [6-8].

In Slovenia, notification of TBE is mandatory and based on the European Union (EU) standardised case definition [9]. Only cases with central nervous system involvement (meningoencephalitic TBE) and laboratory confirmation are notified. Slovenia is one of the countries with the highest notified incidence in Europe, ranging from 8 to 15 per 100,000 in the period from 2009 to 2013, with cases occurring throughout the country [10]. Data for the past 20 years show a non-homogenous age distribution with higher incidence in older age groups (> 40 years) [10]. Preventive measures include the use of repellents, appropriate clothing and daily inspection of the skin to remove ticks [11]. The most effective method of preventing TBE is vaccination [11-13]. Mandatory vaccination against TBE was introduced in Slovenia in 1986 for those at risk of occupational exposure, and in 1990 for students at risk of exposure during curricular training, while the rest of the population needs to pay for the vaccination themselves. TBE vaccination coverage in Slovenia remains low: by 2007, the proportion of the general population reporting to ever have been vaccinated against TBE was 12.4% [14].

In a context where limited resources prevent universal TBE vaccination free of charge, data are needed to identify those groups most affected by the disease so that vaccination can be targeted in order to yield the greatest benefit on population health. Countries have used incidence data to guide vaccination strategies towards specific age groups and geographical areas [15-17]. Estimation of the TBE burden in the form of disability-adjusted life years (DALYs), a summary measure of population health, is better suited to express the overall and age group-specific impact of the disease in the population while taking into account the effects of acute illness and its sequelae on mortality and morbidity [18]. The objective of this study was to estimate the overall and age- and sex-specific annual burden of TBE in Slovenia in order to inform vaccination policy in a setting with limited resources.

## Methods

### Model

To estimate the burden of TBE we used the pathogen-based incidence approach developed by the European Centre for Disease Prevention and Control (ECDC) *Burden of communicable diseases in Europe* project (BCoDE) [18-20]. The burden was expressed in DALYs. DALYs have two components: years of life lost due to premature death (YLL) and healthy years of life lost due to disability (YLD) [21].

**Figure 1:**
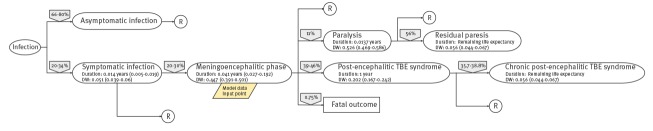
Outcome tree for tick-borne encephalitis virus infection

DW: disability weight; R: resolution of infection; TBE: tick-borne encephalitis.

We used a disease model (outcome tree) based on the current knowledge of the disease progression pathway, linking all health outcomes related to TBE with the initial infection. Starting with the infection a case moved through the outcome tree transitioning into different health outcomes according to different conditional transition probabilities (i.e. probability of occurrence of each health outcome), exiting the tree with a resolved infection, with a life-long disability or with a fatal outcome. In order to measure YLL, life expectancy was based on the standard reference life table developed within the *Global Burden of Disease 2010* project [22]. To measure YLD, each health outcome was characterised by a disease duration and a disability weight. Disability weights quantify health losses to reflect the disability experienced by someone living with a health issue. Based on the severity of the disease, they range from 0 (full health) to 1 (death). The disability weights were generated for BCoDE and the *Global Burden of Disease study* (GBD) 2013 through elicitation methods [23,24]. The outcome tree for TBE used in our model (Figure 1) was based on a thorough review of published studies and on the opinion of ECDC experts [25]. All parameters included in the outcome tree, conditional transition probabilities, durations and disability weights were based on published studies and entailed a certain level of uncertainty. The uncertainty was modelled by incorporating ranges using either uniform or Pert distributions [26] and quantified by performing Monte Carlo simulations with 10,000 iterations to obtain 95% uncertainty intervals (UI). In order to assess age groups of interest for vaccination strategies, we compared the median DALYs and their 95% UIs.

### Input data

The ECDC BCoDE toolkit was used for DALY estimation [25]. Input data for the model were the mean annual numbers of meningoencephalitic TBE cases notified to the Slovenian national surveillance system for communicable diseases from 2009 to 2013. They were stratified by 5-year age groups and by sex. For those calculations where a population estimate was required, we used the 2011 population data for Slovenia obtained from Eurostat [27]. The main type of input data for TBE in the BCoDE toolkit was the number of symptomatic infections (first phase of the disease); to obtain this, surveillance data were multiplied by the appropriate transitional probabilities as specified by the TBE outcome tree. No time discounting was applied, thus future and present disabilities were weighted equally.

## Results

**Figure 2:**
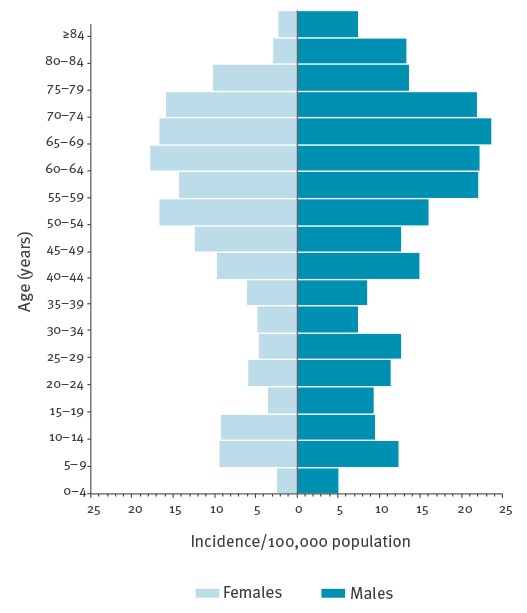
Mean annual incidence per100,000 of tick-borne encephalitis, by age and sex, Slovenia, 2009–2013 (n = 1,190)

From 2009 to 2013, a total of 1,190 cases (58% males) of TBE in their meningoencephalitic phase were notified in Slovenia, with a mean of 238 cases/year. The median age at diagnosis was 51 years (range: 1–86 years). The mean annual incidence of meningoencephalitic TBE was 11.6 per 100,000 population (9.6/100,000 for females and 13.6/100,000 for males). Incidence was higher in older individuals (50–74 years: 18.5/100,000) than in children (5–14 years: 10.2/100,000). Data by 5-year age groups and by sex are presented in Figure 2.

The estimated DALYs per year were 224.52 (95% UI: 210.14-238.84), corresponding to 10.95 DALYs per 100,000 per year (95% UI: 10.25-11.65). Each case of TBE accounted for an average of 0.23 DALYs (95% UI: 0.22–0.24) In the Table, DALYs and their components (YLL and YLD) are presented for all health outcomes related to TBE. YLDs per year accounted for 67% of the total disease burden. Late sequelae, following the meningoencephalitic phase of the disease, contributed to 63% of the DALYs per year.

**Table:** Tick-borne encephalitis annual burden estimates, Slovenia, 2009–2013

	DALYs/year (95% UI)	DALYs/100,000 (95% UI)	YLL/year (95% UI)	YLD/year (95% UI)
Symptomatic infection	0.67 (0.61–0.73)	0.03 (0.03–0.04)	0	0.67 (0.61–0.73)
Meningoencephalitic phase	81.94 (76.77–87.15)	4.00 (3.74–4.25)	74.88 (70.14–79.56)	7.06 (5.92–8.36)
Post-encephalitic TBE syndrome	21.36 (19.87–22.91)	1.04 (0.97–1.12)	0	21.36 (19.87–22.91)
Paralysis	0.20 (0.18–0.21)	< 0.001	0	0.20 (0.18–0.21)
Residual paresis	34.32 (31.98–36.73)	1.67 (1.56–1.79)	0	34.32 (31.98–36.73)
Chronic post-encephalitic TBE syndrome	86.04 (79.87–92.31)	4.20 (3.90–4.50)	0	86.04 (79.87–92.31)
**Total**	**224.52** **(210.14–238.84)**	**10.95** **(10.25–11.65)**	**74.88** **(70.14–79.56)**	**149.64** **(139.67**–**159.75)**

DALYs: disability-adjusted life years; TBE: tick-borne encephalitis; UI: uncertainty interval; YLD: healthy years of life lost due to disability; YLL: years of life lost.

The group of 50–54-year-old women and the group of 25–29-year-old men had the highest point estimates of DALYs per year with 10.56 (95% UI: 7.34–14.03) and 13.02 (95% UI: 9.25–17.49) DALYs per year respectively. When looking at both sexes together, the 50–54 and 55–59-year-olds accounted for the highest number of DALYs, 21.08 (95% UI: 14.91–28.40) and 20.48 (95% UI: 14.48–27.70), respectively.

**Figure 3:**
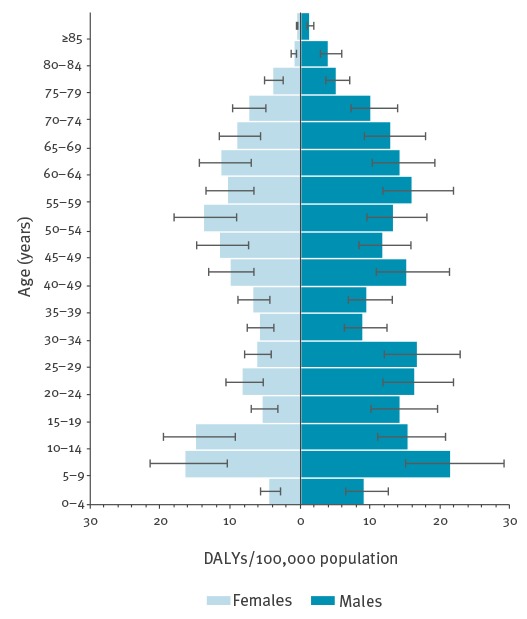
Estimated mean annual disability-adjusted life years per 100,000 stratum-specific population due to tick-borne encephalitis, by age and sex, Slovenia, 2009–2013

In terms of DALYs per 100,000 stratum-specific population, the highest burden point estimate was among the 5–9-year-olds: 19.29 DALYs per 100,000 stratum-specific population per year (95% UI: 15.41–23.90) with 16.62 DALYs (95% UI: 11.48–22.51) and 21.69 DALYs per 100,000 per year (95% UI: 15.12–29.28) for girls and boys, respectively. Data by 5-year age groups and by sex are presented in Figure 3.

DALYs: disability-adjusted life years.

The whiskers represent 95% uncertainty intervals.

**Figure 4:**
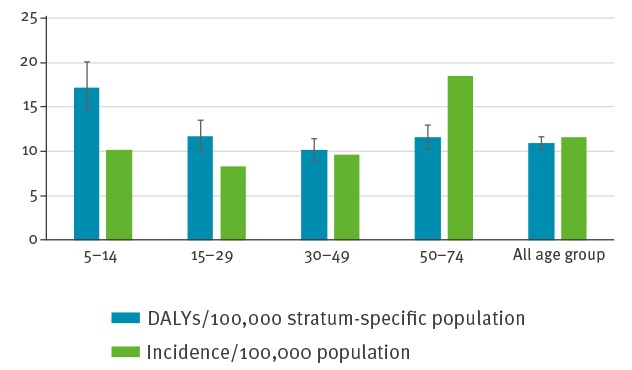
Estimated mean annual incidence per 100,000 and mean annual disability-adjusted life years per 100,000 stratum-specific population due to tick-borne encephalitis, by age group, Slovenia, 2009–2013

The group of 50–74-year-olds had a lower TBE burden estimate of 11.58 (95% UI: 10.25–12.91) DALYs per 100,000 stratum-specific population per year in comparison to the 5–14-year-olds with a burden of 17.31 (95% UI: 14.58–20.08) DALYs per 100,000 stratum-specific population per year (Figure 4).

DALYs: disability-adjusted life years.

The whiskers represent 95% uncertainty intervals.

## Discussion

In this paper we present the overall and the age- and sex-specific annual burden of TBE in Slovenia expressed in DALYs. The use of DALYs integrates mortality and morbidity from TBE in a single composite health metric, giving a comprehensive estimate of the impact of this disease on population health.

An analysis of notified TBE cases in the 5-year period from 2009 to 2013 confirms Slovenia as one of the countries, together with the Baltic states and the Russian Federation, where reported incidence per 100,000 is the highest in Europe [11,28]. With an estimate of 10.95 DALYs per 100,000 per year (95% UI: 10.25-11.65), TBE has an important impact on the health of the Slovenian population. In accordance with input incidence data, we found consistently higher burden point estimates in male persons across all ages. According to the BCoDE 2009–13 study, the estimated burden of TBE in Slovenia was nine times higher than the corresponding estimated burden of TBE measured in DALYs per 100,000 population per year for the EU and European Economic Area (EEA) for the same time period [29]. Moreover, the impact of TBE on the Slovenian population is comparable to that of healthcare-associated neonatal sepsis (16.8 DALYs/100,000) according to a recent study on healthcare-associated infection in the EU/EEA [30].

Looking at incidence data alone, older age groups (50–74-year-olds) appeared most affected by TBE in Slovenia. However, the use of DALYs identified children (5–14-year-olds) as the group with a higher burden. This difference in impact of TBE would not have been detected, if we had limited our assessment to incidence data, ignoring the combined effects of morbidity, short- and long-term sequelae and mortality. Other countries with a similar TBE incidence profile as Slovenia could profit from this approach to identify groups with important burden, particularly when informing decision makers about the allocation of limited resources for targeted public health interventions (i.e. vaccination). 

Vaccination is regarded as the most effective preventive measure for TBE [11]. Studies have shown a 96–99% field effectiveness in persons receiving three doses following the recommended schedule [12,13]. In neighbouring Austria, an estimated 88% of the general population are vaccinated with at least one dose, while 58% are vaccinated regularly following the advised schedule [13]. Austria has managed to reduce the number of TBE cases by 90% by increasing its vaccination rate from 6% in 1980 to its current level [13]. Despite the fact that vaccination has been recommended in Slovenia for decades, only 12% of the population was vaccinated with at least one dose by 2007 and only 7.3% get vaccinated regularly following the advised schedule [31]. 

TBE vaccination remains a self-paid expense for the majority of the population. The costs are covered by the mandatory insurance system or by the employer only in case of occupational exposure or exposure during education or training. Data from 2007 show that only 4.6% of the population paid themselves for TBE vaccination [14]. A recent study from Šmit et al., estimating DALYs of TBE in Slovenia using the GBD project methodological approach, supports the need for a public health strategy aimed at increasing the national vaccination coverage [32]. Multiple factors influencing the decision to get vaccinated against TBE (knowledge, trust, accessibility, cost) should be considered when planning strategies aimed at increasing vaccination coverage [33]. Projections, however, show that the impact of a vaccine subsidy, making the vaccine free of charge, could alone increase coverage by 45%, and even more in low-income households [34]. 

Increasing TBE vaccination coverage should be considered as an option for intervention to reduce the impact of TBE [10,32]. In the presence of limited resources, the implementation of such a measure could be difficult in the short term. Our results suggest that effective prevention of TBE in children would have the highest impact in terms of DALYs of TBE averted. This novel insight in the distribution of TBE burden should be considered when prioritising access to TBE vaccination and could improve previous recommendations originating from incidence data alone, where the focus was mainly on older age groups [10]. 

Prioritising vaccination in children could be easier thanks to the well-functioning Slovenian national childhood immunisation programme. It is also important to take into account the need for booster doses of the TBE vaccine. In the age groups of interest, a three-dose primary vaccination schedule with a first booster dose after 3 years and further boosters every 5 years is recommended to maintain seropositivity [35]. A recent study showed that a schedule that includes the first booster dose yields a high and long-lasting (> 5 years) immune response, thus suggesting that subsequent TBE booster intervals could be extended beyond the current recommendation [36]. Considering the financial implications of lifelong booster doses (and the different schedules that apply at different ages), age-specific cost-effectiveness studies are needed to inform decisions on the extent to which TBE vaccine can be subsidised in order to achieve the highest level of immunopersistence and impact on TBE burden in a cost-effective manner.

We considered prioritising the most affected areas or regions as an alternative approach. Although some regions in Slovenia are more affected then others, TBE occurs throughout the country. Considering the epidemiological situation of TBE in Slovenia, the country`s relatively small area and population size, as well as the mobility of the population between regions, we consider this approach could be potentially misleading and lead to health inequalities. Other countries where restricted areas or regions are affected could consider a modelling approach stratified by region.

This study has certain limitations. The outcome tree describing the progression pathway of the disease assumes no differences in disease progression between different age groups. Lifelong sequelae make an important contribution to the overall burden, especially in the younger age groups. The disease in children is commonly regarded as mild, but evidence is increasing for the relevance of severe acute disease and long-term sequelae of TBE in children, as well as for the lack of knowledge around the matter [5,37-46]. The uncertainty around the disease progression, overall and for different age groups, can lead to an over- or underestimation of the burden overall and in different age groups. Future study of the disease progression of TBE in different age groups is needed and could improve the accuracy of the model. Another limitation of our study is that the data set used for input in the model was not corrected for underestimation (due to under-reporting and under-ascertainment) of the surveillance system [47]. At the moment of writing, data on underestimation of TBE notification were not available. However, taking into consideration the structure of the morbidity surveillance pyramid [47], we can assume that the notified data were still underestimating the true incidence of disease, thus leading to an underestimation of our burden estimates.

DALYs are a composite health metric highly dependent on the assumptions made; it is commonly used for ranking the relative burden of diseases within the same study, in cost-effectiveness analyses or evaluations of interventions (e.g. DALYs averted). The differences in absolute values between our results and the recent study from Šmit et al. [32] are probably due to differences in underlying assumptions and disease modelling approaches. Šmit et al. used data from a single year that had more cases than the 5-year annual average we used; they used an underestimation coefficient (4.5) for the number of cases of meningoencephalitic TBE, but we did not find enough evidence to make such assumptions; they modelled all neurological sequelae as lifelong. Moreover, Šmit et al. used higher transitional probabilities (in the age groups older than 15 years) and higher disability weights when modelling mild sequelae. Taking this into consideration, a direct comparison is not valid. Our focus on the distribution of the TBE burden across different age groups enabled us to suggest efficient options for vaccination.

## Conclusion

We identified a higher burden of TBE among children aged 5–14 years than among adults aged 50–74 years despite a lower TBE incidence. Incidence data alone do not fully reflect the disease impact and should not be the only indicator to inform vaccination policy. In a limited resource setting where prioritisation of TBE vaccination strategies is required, vaccination programmes targeting children should be considered as possibly having a higher impact on disease burden. Our data could be used for future cost-effectiveness studies.
